# Effects of different vitamins on individuals with septic shock: a Bayesian NMA of RCTs

**DOI:** 10.3389/fnut.2025.1566422

**Published:** 2025-08-13

**Authors:** Jinjin Tian, Ling Long, Dandan Li, Yahan Liang, Guinan Sun, Wenjing Song, Xizi Yue, Limin Shen, Heling Zhao, Shan Ren

**Affiliations:** ^1^Department of Intensive Care Medicine, Hebei General Hospital, Hebei Medical University, Shijiazhuang, Hebei, China; ^2^Department of Intensive Care Medicine, Hebei General Hospital, Shijiazhuang, Hebei, China; ^3^Department of Intensive Care Medicine, Hebei General Hospital, North China University of Science and Technology, Shijiazhuang, Hebei, China

**Keywords:** vitamins, septic shock, network meta-analysis, vitamin D, shock

## Abstract

**Objective:**

To compare the effects of different vitamins on patients with septic shock (SS) through Bayesian network meta-analysis.

**Methods:**

Randomized controlled trials (RCTs) on vitamins for septic shock patients were retrieved from PubMed, Embase, Cochrane Library, Web of Science, etc. The retrieval time was set from the establishment of the database to May 20, 2024. All relevant studies on vitamin treatment for septic shock were retrieved and screened according to the established inclusion and exclusion criteria. Intensive care unit (ICU) length of stay, mechanical ventilation time, Sequential Organ Failure Assessment (SOFA) scores after 24 h, total hospital stay, and 28-day mortality were used as outcome measures. The quality of the included studies was evaluated for risk of bias, and R software was used for data analysis.

**Results:**

A total of 36 articles were included in the analysis, covering 4,473 patients with septic shock. The vitamins included vitamin B (VB), vitamin C (VC), vitamin D (VD), vitamin E (VE), hydroxocobalamin (HYD), and vitamin combinations such as hydrocortisone plus vitamin C plus vitamin B (HYDVCVB), vitamin D plus probiotics (VDP), vitamin C plus vitamin B (VCVB), and hydrocortisone plus vitamin C (HYDVC). The network meta-analysis results showed that in terms of ICU length of stay, VD was superior to the control group [mean difference (MD) = 4.57, 95% CI (1.01, 9.69)] and HYDVCVB [MD = 5.4, 95% CI (0.51, 11.66)], with statistically significant differences. In terms of mechanical ventilation time, VC, VD, VCVB, and HYDVCVB showed no statistically significant differences compared to the control group. Regarding the SOFA score after 24 h, VDP was superior to the control group [MD = 2.98, 95% CI (0.27, 5.62)], as well as HYDVCVB [MD = 3.32, 95% CI (0.59, 6.04)], VB [MD = 2.96, 95% CI (0.18, 5.67)], VC [MD = 2.91, 95% CI (0.17, 5.57)], VCVB [MD = 3.18, 95% CI (0.31, 5.9)], and VD [MD = 2.91, 95% CI (0.05, 5.71)], with statistically significant differences. In terms of total hospital stay, VD was superior to the control group [MD = 7.61, 95% CI (2.59, 12.63)], as well as HYDVCVB [MD = 7.71, 95% CI (2.55, 12.9)], VB [MD = 7.6, 95% CI (0.84, 14.39)], VC [MD = 9.93, 95% CI (3.9, 15.92)], and VCVB [MD = 8.1, 95% CI (1.79, 14.41)], with statistically significant differences. Regarding 28-day mortality, VB, VC, VD, VDP, VCVB, HYDVCVB showed no statistically significant differences compared to the control group.

**Conclusion:**

In patients with septic shock, the use of VD shows certain advantages in reducing ICU length of stay and total hospital length of stay. Moreover, its combination with probiotics may help reduce the SOFA scores after 24 h. However, these interventions have not significantly impacted 28-day mortality or mechanical ventilation time.

**Systematic review registration:**

https://www.crd.york.ac.uk/prospero/, PROSPERO: CRD42024599094.

## 1 Introduction

Sepsis is a severe inflammatory response syndrome that is triggered by an excessive immune response to infection and is a life-threatening organ dysfunction (1). It can be identified by an acute change in the Sepsis-related Organ Failure Assessment (SOFA) score of at least 2 points following infection. Septic shock (SS) is defined as a subset of sepsis characterized by circulatory, cellular, and metabolic abnormalities, which are associated with a higher risk of death. Clinically, it is defined as patients who meet the diagnostic criteria for sepsis and who, despite adequate fluid resuscitation, require vasopressors to maintain a mean arterial pressure of at least 65 mmHg and have a lactate level >2 mmol/L (2). Despite years of research and therapeutic advancements, sepsis and septic shock remain one of the most common reasons for intensive care unit (ICU) admissions, posing a significant burden on the healthcare system, with 18.6 cases per 1,000 hospital admissions related to this condition, and a mortality rate exceeding 50% in patients with SS (3–5). Moreover, the higher incidence of sepsis in resource-limited settings further underscores the necessity for ongoing efforts to enhance prevention, clinical recognition, and treatment to reduce the global burden of this disease (2, 6, 7).

Restoring hemodynamic stability and therapeutic options for treating critically ill patients with sepsis or septic shock include fluid resuscitation, antimicrobial therapy, vasopressors, mechanical ventilation, and adjunctive metabolic therapy, which may involve the use of vitamin C (VC), thiamine, and corticosteroids either alone or in various combinations (8–10). Vitamins are essential micronutrients that play a key role in many biological pathways associated with sepsis, including those leading to anti-inflammatory and antioxidant effects (11, 12). In addition, relative vitamin deficiency in plasma is common during sepsis, and vitamin therapy has been associated with improved outcomes in some observational and randomized controlled trials (RCTs) involving adult and pediatric sepsis patients (13–16). The biological plausibility and supportive clinical evidence for some major vitamins [such as vitamin C, thiamine, and vitamin D (VD)] form a strong argument for their use in sepsis. However, to date, the results of vitamin supplementation in large multicenter randomized controlled trials (RCTs) and observational studies have been inconsistent. Overall, the evidence for the role of vitamins in sepsis remains mixed.

Therefore, we conducted a meta-analysis to analyze the evidence from randomized controlled trials to assess the efficacy and safety of different vitamins in critically ill adult and pediatric patients, particularly those with septic shock.

## 2 Methods and data

### 2.1 Methods

The NMA adhered to the guidelines outlined in the Preferred Reporting Items for Systematic Reviews and Meta-Analyses (PRISMA) (17) and was registered on the International Prospective Register of Systematic Reviews (PROSPERO), with the registration No. of CRD42024599094.

### 2.2 Literature retrieval

RCTs investigating the role of various vitamins on individuals with SS were retrieved in Cochrane, PubMed, Embase and Web of Science spanning from the establishment of each database to May 2024. The search strategy incorporated a blend of MeSH and free-text terms associated with vitamins and SS. The comprehensive search strategy can be found in Supplementary material S1.

### 2.3 Inclusion and exclusion criteria

Adults and children who met the diagnostic criteria for SS in the Third International Consensus Definitions for Sepsis and Septic Shock (13) revised in 2016 were included. The vitamins included vitamin B (VB), vitamin C (VC), vitamin D (VD), vitamin E (VE), hydroxocobalamin (HYD), and vitamin combinations such as hydrocortisone plus vitamin C plus vitamin B (HYDVCVB), vitamin D plus probiotics (VDP), vitamin C plus vitamin B (VCVB), and hydrocortisone plus vitamin C (HYDVC), while the control group adopted placebo. The primary outcomes assessed encompassed hospital stay duration, 28-day death rate, SOFA scores after 24 h, mechanical ventilation duration, and ICU stay. The study type was RCT.

Duplicates, animal experiments, case explorations, meeting abstracts, reviews, articles with unavailable full texts, and studies involving participants with other organ comorbidities were ruled out.

### 2.4 Data retrieval

Two reviewers autonomously retrieved the articles based on the pre-given criteria. Any discrepancies were addressed through discussion or consulting with a third party to reach an agreement. Information gathered from the selected articles encompassed essential elements like the primary author, publication year, region, sample size, sex, average age, interventions adopted, and outcome metrics.

### 2.5 Quality assessment

Two investigators autonomously evaluated the risk of bias as low, unclear, or high utilizing the tools provided by Cochrane Collaboration (18). In instances of disagreement, a third party was engaged to help reach an agreement. The evaluation encompassed seven domains, including random sequence generation (selection bias), concealment of allocation (selection bias), blinding of personnel and participants (implementation bias), blinding of outcome assessors (detection bias), completeness of outcome data (follow-up bias), selective outcome reporting (reporting bias), and other possible sources of bias. Every enrolled research was individually assessed based on these criteria. Studies meeting all criteria were deemed “low risk” of bias, indicative of high quality and negligible overall bias. Studies that partially fulfilled the criteria were labeled as “unclear risk,” indicating a moderate likelihood of bias. Studies that did not meet any criteria were designated as “high risk,” indicating elevated bias risk and diminished study quality.

### 2.6 Data analysis

An NMA was conducted utilizing a prior vague random effects model with the R 4.4.1 (R Foundation for Statistical Computing). A Markov Chain Monte Carlo (MCMC) technique was utilized (19) to derive the optimal combined estimate and probabilities associated with each protocol. Continuous findings were presented as the posterior mean difference (MD) accompanied by the respective 95% confidence interval (95% CI). The combined effect indicators of binary variables were represented by odds ratio (OR) and 95% CI. The Surface Under the Cumulative Ranking curve (SUCRA) percentages were computed to evaluate the probability of each approach being the most favorable. Network and funnel graphs were visualized utilizing Stata (v15.0) with an incorporated metan command. Within the network diagrams, individual nodes represented medications, with the connections illustrating the comparisons made between them. The size of each circle had a positive correlation with the sample size (number of patients enrolled). Cumulative probability graphs were visualized utilizing the ggplot 2 package.

## 3 Results

### 3.1 Data screening and findings

**Figure 1:**
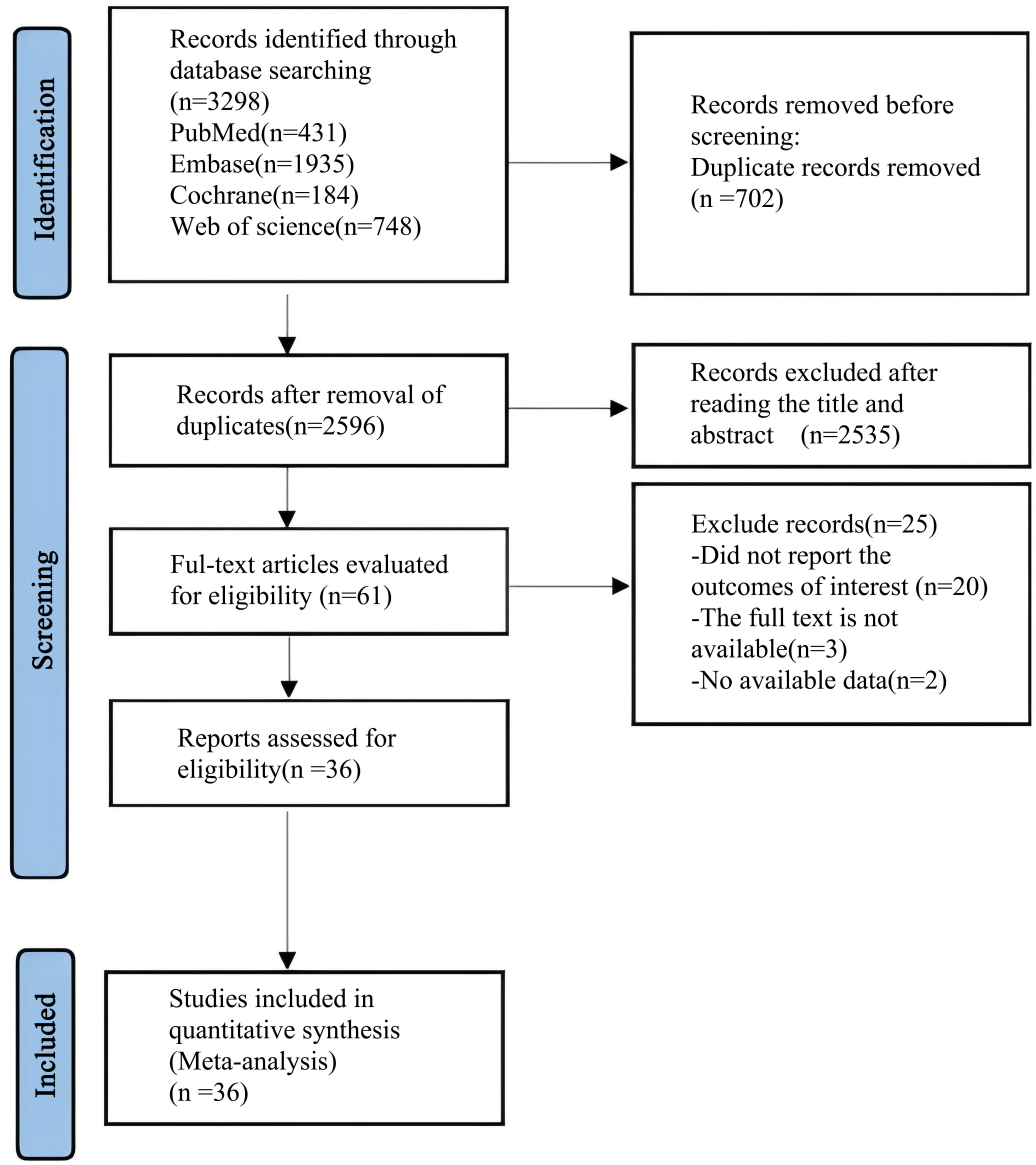
PRISMA flow diagram of the study process.

A preliminary search of the databases yielded 3,298 articles. After removing 702 duplicates, 2,535 articles were excluded based on the review of titles and abstracts. A further 25 articles were excluded after full-text review, leaving a final total of 36 articles (20–54) for analysis. The process of literature screening is shown in Figure 1: flowchart of literature search.

### 3.2 Basic characteristics of articles and risk of bias evaluation

**Figure 2:**
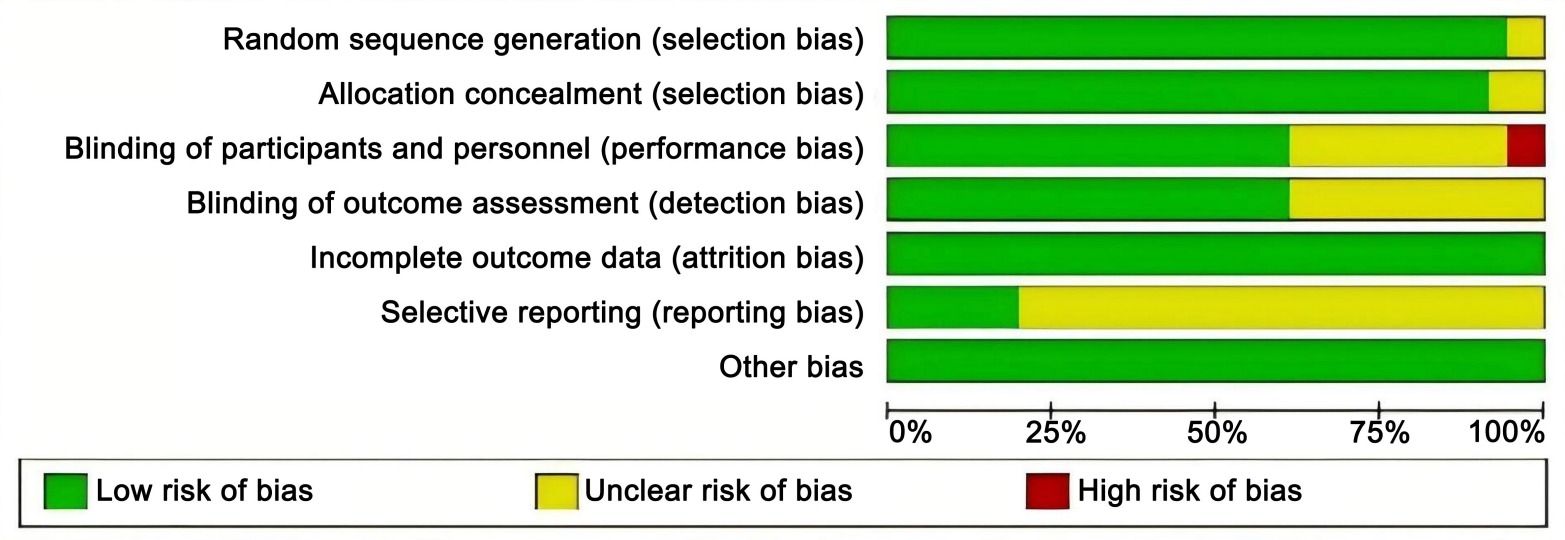
Risk of bias graph.

Altogether 36 articles (20–54) were included in the analysis, involving 4,473 patients with SS. Vitamins included VB, VC, VD, VE, HYD, and vitamin combinations included HYDVCVB, VDP, VCVB, and HYDVC. Table 1 outlines the characteristics of the articles. The blinding method applied in the included articles was clearly explained, and the high risk was mainly caused by deviations in planned interventions. The assessment of the risk of bias in the enrolled articles is depicted in Figure 2.

**Table 1:** Characteristics of eligible studies that were included in the systematic review.

**Study**	**Year**	**Country**	**Sample size**	**Gender (M/F)**	**Mean age (years)**	**Intervention**	**Outcome**
1.Luregn	2024	Australia	HYDVCVB: 27 Control: 33	31/29	HYDVCVB: 4.4 Control: 5.7	HYDVCVB: hydrocortisone, 1 mg/kg; vitamin C, 30 mg/kg; vitamin B, 4 mg/kg	F6; F11
2.Li	2024	China	VC: 20 VC: 20 Control: 18	42/16	VC: 72 VC: 63.3 Control: 67.78	VC: vitamin C, 150 mg/kg/d; VC: vitamin C, 50 mg/kg/d	F11; F15
3.Alfredo	2023	Mxico City	VC: 25 VE: 27 Control: 29	70/61	VC: 62 VE: 70 Control: 75	VC: vitamin C, 1,000 mg; VE: vitamin E, capsules a-tocopherol of 400 IU	F15
4.Adham	2023	Qatar	HYDVCVB: 53 Control: 53	75/31	HYDVCVB: 49.2 Control: 49.1	HYDVCVB: hydrocortisone, 50 mg; Vitamin C, 1.5 mg; vitamin B, 200 mg	F6; F15; F17
5.Ari	2023	America	VB: 42 Control: 46	63/25	VB: 42 Control: 46	VB: vitamin B, 200 mg	F15
6.Fumitaka	2023	Australia	VC: 15 Control: 15	21/9	VC: 64 Control: 70	VC: Vitamin C, 3,000 mg	F15
7.Wang	2023	China	HYDVCVB: 12 Control: 10	15/7	HYDVCVB: 56 Control: 59	HYDVCVB: Hydrocortisone, 200 mg; Vitamin C, 1,500 mg; Vitamin B, 200 mg	F6; F11; F42
8.David	2022	America	VC: 60 Control: 64	63/61	VC: 68.9 Control: 73	VC: Vitamin C, 1,000 mg	F6; F11; F15; F17; F42
9.Lyu	2022	China	HYDVCVB: 213 Control: 213	285/141	HYDVCVB: 69.0 Control: 71.0	HYDVCVB: Hydrocortisone, 200 mg; Vitamin C, 2,000 mg; vitamin B, 200 mg	F6; F11; F15; F17; F42
10.Nandhini	2022	India	VB: 25 VC: 25 Control: 25	38/37	VB: 53.68 VC: 49.96 Control: 53.64	VB: vitamin B, 2 mg/kg VC: vitamin C, 25 mg/kg	F15
11. Patrice	2022	New Zealand	VC: 20 Control: 20	27/13	VC: 69 Control: 69	VC: vitamin C, 25 mg/kg	F6; F15
12. Lamontagne	2022	Canada	VC: 429 Control: 433	538/324	VC: 65 Control: 65.2	VC: vitamin C, 50 mg/kg	F11; F15
13. Jayshil	2022	American	HYD: 10 Control: 10	10/10	HYD: 64 Control: 57	HYD: hydroxocobalamin, 5,000 mg	F11
14. Arun	2021	India	HYDVCVB: 30 Control: 30	38/22	HYDVCVB: 36.7 Control: 37.5	HYDVCVB: hydrocortisone, 50 mg; vitamin C, 6,000 mg/day; vitamin B, 200 mg 12 hourly	F15; F42
15. Noha	2021	Egypt	VDP: 20 VCVB: 20 Control: 20	52/8	VDP: 44.95 VCVB: 42.15 Control: 48.75	VDP: vitamin D 400,000 IU of vitamin D3 plus probiotics; VCVB: vitamin C 1,000 mg plus vitamin B1 200 mg	F11; F15
16. Abdelrhman	2021	Egypt	HYDVCVB: 47 Control: 47	41/53	HYDVCVB: 65.81 Control: 61.60	HYD: hydrocortisone, 50 mg/6 h; vitamin C, 1,500 mg/6 h; vitamin B, 200 mg/12 h	F11; F17
17. Jonathan	2021	America	HYDVCVB: 252 Control: 249	273/228	HYDVCVB: 62 Control: 61	HYDVCVB: hydrocortisone, 50 mg; vitamin C, 1,500 mg; vitamin B, 100 mg	F15
18. Mohammad	2021	Iran	HYDVC: 29 Control: 29	47/11	HYDVC: 45.4 Control: 45.4	HYDVC: hydrocortisone, 50 mg/6 h; Vitamin C, 1,500 mg/6 h	F15
19. Gayathri	2021	India	VC: 20 VB: 20 VCVB: 20 Control: 20	68/32	/	VC: Vitamin C, 2,000 mg; VB: vitamin B, 200 mg; VCVB: vitamin C, 2,000 mg, vitamin B, 200 mg	F15
20. Jose	2020	America	HYDVCVB: 68 Control: 69	59/78	HYDVCVB: 70 Control: 67	HYDVCVB: Hydrocortisone, 50 mg q6h; Vitamin C, 1,500 mg; vitamin B, 200 mg	F6; F15
21. Sung	2020	Korea	VCVB: 53 Control: 58	42/69	VCVB: 70 Control: 69	VCVB: Vitamin C, 50 mg/kg; vitamin B, 200 mg	F6; F15; F17F11
22. Lv	2020	China	VC: 61 Control: 56	59/58	VC: 58.7 Control: 60.2	VC: Vitamin C, 3,000 mg	F11; F15
23. Suttasinee	2020	Thailand	VB: 25 Control: 25	29/21	VB: 64 Control: 66	VB: vitamin B, 200 mg	F11; F15
24. Wang yu	2020	China	VD: 55 Control: 54	64/45	VD: 3.9 Control: 4.2	VD: 50,000 IU of vitamin D	F15; F17
25. Tomoko	2020	Australia	HYDVCVB: 107 Control: 104	133/78	HYDVCVB: 61.9 Control: 61.6	HYDVCVB: Hydrocortisone 50 mg; Vitamin C 1,500 mg; vitamin B 200 mg	F6; F11; F15
26. Moskowitz	2020	America	HYDVCVB: 101 Control: 99	111/89	HYDVCVB: 68.9 Control: 67.7	HYDVCVB: Hydrocortisone 50 mg; Vitamin C 1,500 mg; vitamin B 100 mg	F11; F15
27. Zubair	2020	India	HYDVCVB: 45 Control: 43	63/25	HYDVCVB: 58.69 Control: 59.37	HYDVCVB: Hydrocortisone 50 mg; Vitamin C 1,500 mg; vitamin B 200 mg	F6; F15; F42
28. Saleem	2020	India	HYDVCVB: 50 Control: 50	69/31	HYDVCVB: 65 Control: 70	HYDVCVB: Hydrocortisone: 50 mg q6 hourly for 7 days; Vitamin C: 1,500 mg q6 hourly for 4 days; vitamin B: 200 mg q12 hourly for 4 days	F6; F11; F15
29. Ping	2020	China	HYDVCVB: 40 Control: 40	43/37	HYDVCVB: 59.5 Control: 63.7	HYDVCVB: Hydrocortisone 50 mg every 6 h for 7 days; Vitamin C 1,500 mg every 6 h for 4 days; vitamin B 200 mg every 12 h for 4 days	F11
30. Alfredo	2020	Mexico	VC: 18 VE: 18 Control: 21	49/48	VC: 62 VE: 65.5 Control: 76	VC: vitamin C, 1 mg; VE: vitamin E, 400 UI	F15
31. Hasanali	2019	Iran	VCVB: 50 Control: 50	43/57	VCVB: 56.21 Control: 61.07	VCVB: VC: Vitamin C, 50 mg/kg; vitamin B at a dose of 200 mg	F15
32. Nur	2019	Malaysia	VB: 33 Control: 32	38/27	VB: 67 Control: 63.5	VB: vitamin B, 200 mg for 3 days	F15; F42
33. Ding	2017	China	VD: 29 Control: 28	33/24	VD: 57.4 Control: 56	VD: vitamin D, 300,000 IU	F11; F17; F42
34. Mohadeseh	2016	Iran	VC: 14 Control: 14	21/7	VC: 64.14 Control: 63.71	VC: Vitamin C, 25 mg/kg	F11; F42
35. Michael	2016	America	VB: 43 Control: 45	52/36	VB: 70 Control: 65	VB: vitamin B 200 mg	F15; F6; F42
36. Sadeq	2015	America	VD: 10 VD: 10 Control: 10	18/12	VD: 62 VD: 64 Control: 65	VD: 400,000 IU vitamin D; VD: 200,000 IU vitamin D	F6; F11; F42

VB, vitamin B; VC, vitamin C; VD, vitamin D; VE, vitamin E; HYDVCVB, hydrocortisone plus vitamin C plus vitamin B; HYD, hydroxocobalamin; VDP, vitamin D plus probiotics; VCVB, vitamin C plus vitamin B; HYDVC, hydrocortisone plus vitamin C; F6, Length of stay in hospital; F11, 28-day mortality; F15, Sepsis-related Organ Failure Assessment (SOFA); F17, Mechanical ventilation (MV) duration (days); F42, Intensive care unit (ICU) length of stay.

### 3.3 NMA results

#### 3.3.1 Effect of treatment on ICU length of stay

**Figure 3:**
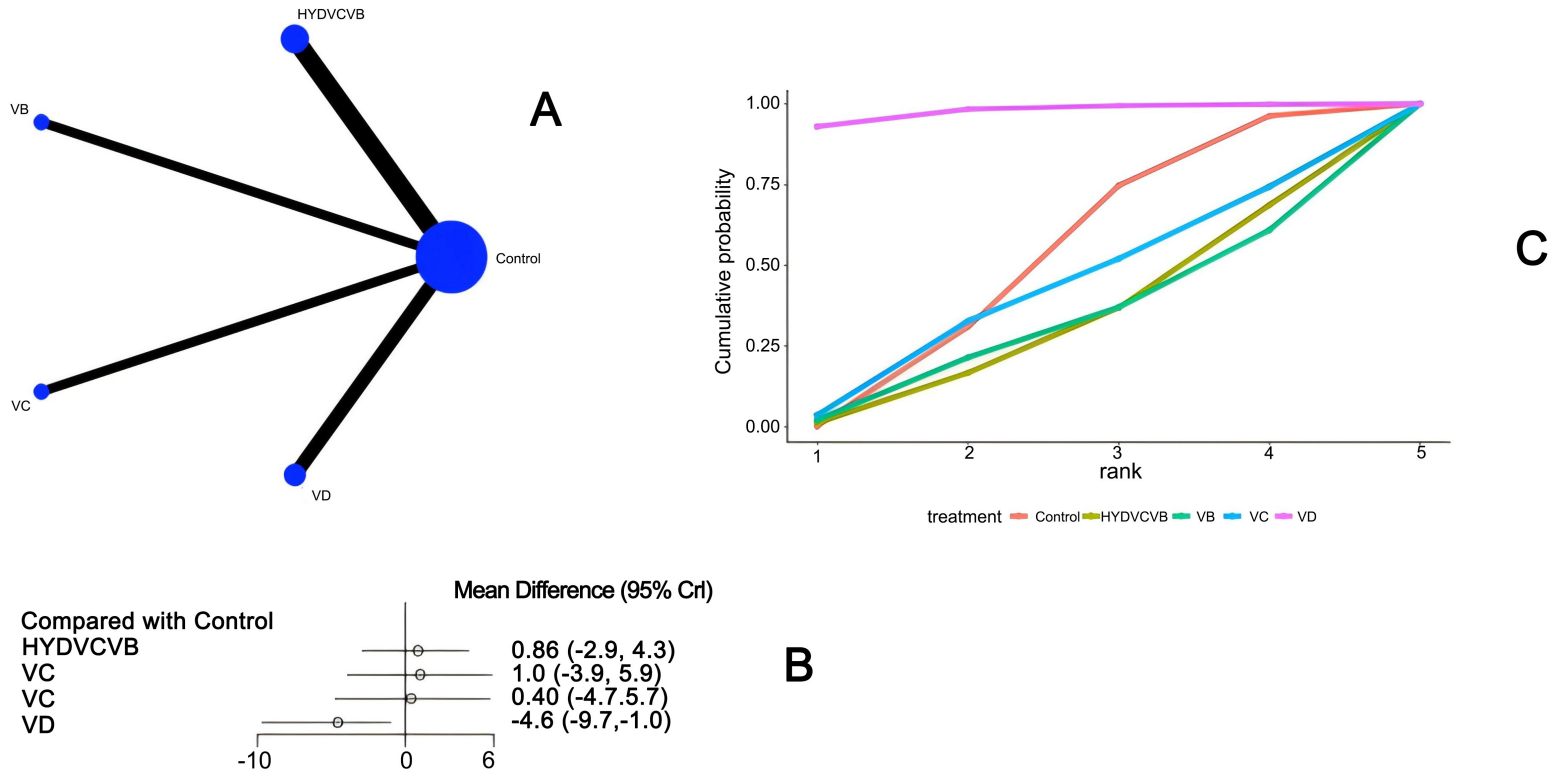
The impact of various vitamins on ICU duration of stay. **(A)** Network diagram illustrating the impact of various vitamins on ICU length of stay. **(B)** Forest plot illustrating the impact of various vitamins on ICU length of stay. **(C)** SUCRA plot illustrating the impact of various vitamins on ICU length of stay.

Ten studies (26–28, 33, 46, 51–54) mentioned the impact of different vitamin treatments on ICU length of stay, as shown in Figure 3. From the network diagram, we found that direct comparisons were formed, among which the number of studies comparing HYDVCVB with the control group was the largest. The network relationship diagram is shown in Figure 3A. In the forest plot, compared with the control group [MD = −4.6, 95% CI (−9.7, −1.0)], VD could reduce the number of days of ICU stay. The forest plot is shown in Figure 3B. In the league table, it is further shown that compared with the control group [MD = 4.57, 95% CI (1.01, 9.69)] and HYDVCVB [MD = 5.4, 95% CI (0.51, 11.66)], VD has an advantage in shortening ICU length of stay, and the differences are all statistically significant. See Supplementary Table S1 for details. According to the Surface Under the Cumulative Ranking Curve (SUCRA) line chart, VD has the largest area, indicating that the effect of VD in shortening ICU length of stay in septic shock may be the best. The SUCRA plot is detailed in Figure 3C.

#### 3.3.2 Effect of treatment on mechanical ventilation duration

**Figure 4:**
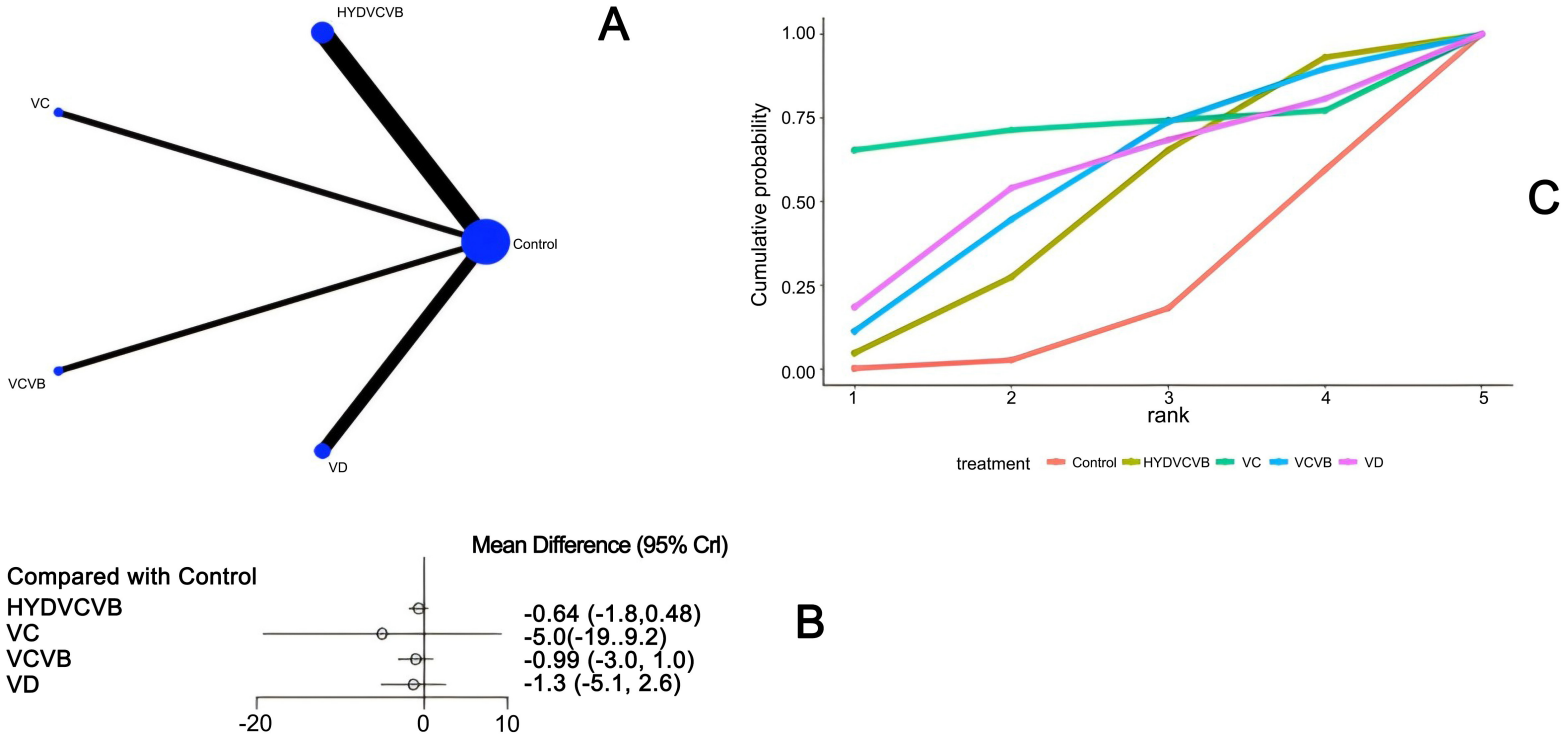
The effect of different vitamins on mechanical ventilation duration. **(A)** Network diagram illustrating the effect of treatment on mechanical ventilation duration. **(B)** Forest plot illustrating the effect of treatment on mechanical ventilation duration. **(C)** SUCRA plot illustrating the effect of treatment on mechanical ventilation duration.

Seven studies (23, 27, 28, 35, 40, 43, 52) have examined the impact of different vitamin treatments on the duration of mechanical ventilation, as shown in Figure 4. The network diagram reveals that direct comparisons were formed, with the most studies comparing HYDVCVB to the control group. The detailed network relationship diagram is presented in Figure 4A. In the forest plot analysis, no significant differences were found when comparing HYDVCVB, VC, VCVB, and VD to the control group. The specific forest plot is detailed in Figure 4B. Furthermore, the league table also indicates that there were no statistically significant differences when comparing HYDVCVB, VC, VCVB, and VD to the control group. The detailed league table is provided in Supplementary Table S2. The Surface Under the Cumulative Ranking Curve (SUCRA) plot is shown in Figure 4C. In summary, the application of HYDVCVB, VC, VCVB, and VD in septic shock does not significantly affect the duration of mechanical ventilation.

#### 3.3.3 Effect of treatment on SOFA score after 24 h

**Figure 5:**
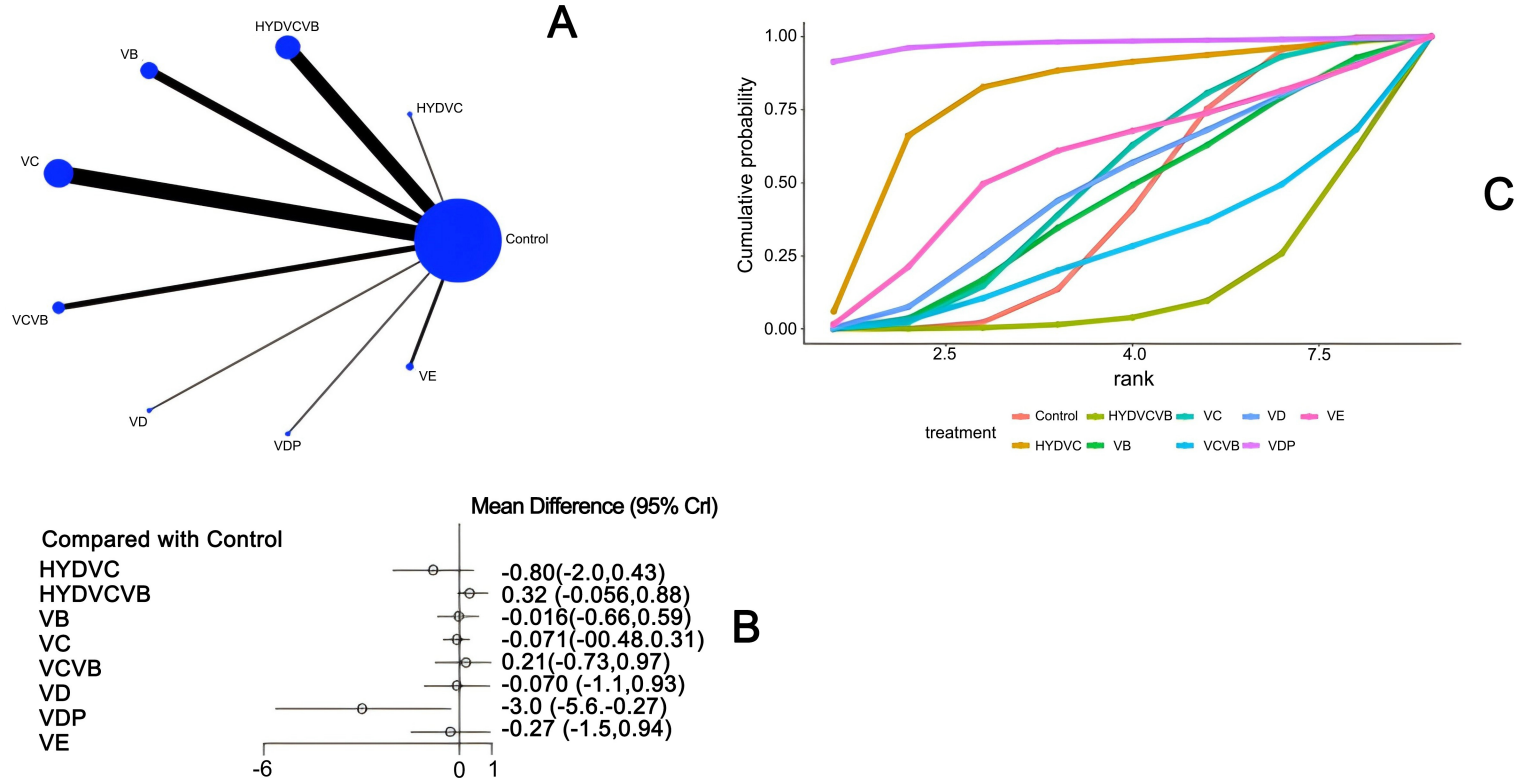
The effect of different vitamins on SOFA scores after 24 h. **(A)** Network diagram illustrating the effect of treatment on SOFA score after 24 h. **(B)** Forest plot illustrating the effect of treatment on SOFA score after 24 h. **(C)** SUCRA plot illustrating the effect of treatment on SOFA score after 24 h.

Twenty-eight studies (21–25, 27–31, 33, 34, 36–47, 49–51) have examined the impact of different vitamins on the Sequential Organ Failure Assessment (SOFA) score 24 h after treatment, as shown in Figure 5. The network diagram reveals that direct comparisons were formed, with the most studies comparing Vitamin C to the control group. The detailed network relationship diagram is presented in Figure 5A. In the forest plot analysis, Vitamin D plus probiotics (VDP) was found to significantly reduce the SOFA score 24 h after treatment when compared to the control group [MD = −3.0, 95% CI (−5.6, −0.27)]. This indicates that VDP can effectively lower the SOFA score. The specific forest plot is detailed in Figure 5B. The league table further highlights that VDP has a significant advantage in reducing the SOFA score 24 h after treatment when compared to the control group [MD = 2.98, 95% CI (0.27, 5.62)], HYDVCVB [MD = 3.32, 95% CI (0.59, 6.04)], vitamin B [MD = 2.96, 95% CI (0.18, 5.67)], vitamin C [MD = 2.91, 95% CI (0.17, 5.57)], VCVB [MD = 3.18, 95% CI (0.31, 5.9)], and vitamin D [MD = 2.91, 95% CI (0.05, 5.71)]. These differences are statistically significant, suggesting that VDP is more effective in reducing the SOFA score. The detailed league table is provided in Supplementary Table S3. The Surface Under the Cumulative Ranking Curve (SUCRA) plot shows that VDP has the largest SUCRA value, indicating that VDP may be the most effective treatment for reducing the SOFA score 24 h after treatment in patients with septic shock. The SUCRA plot is detailed in Figure 5C.

#### 3.3.4 Effect of treatment on total length of hospital stay

**Figure 6:**
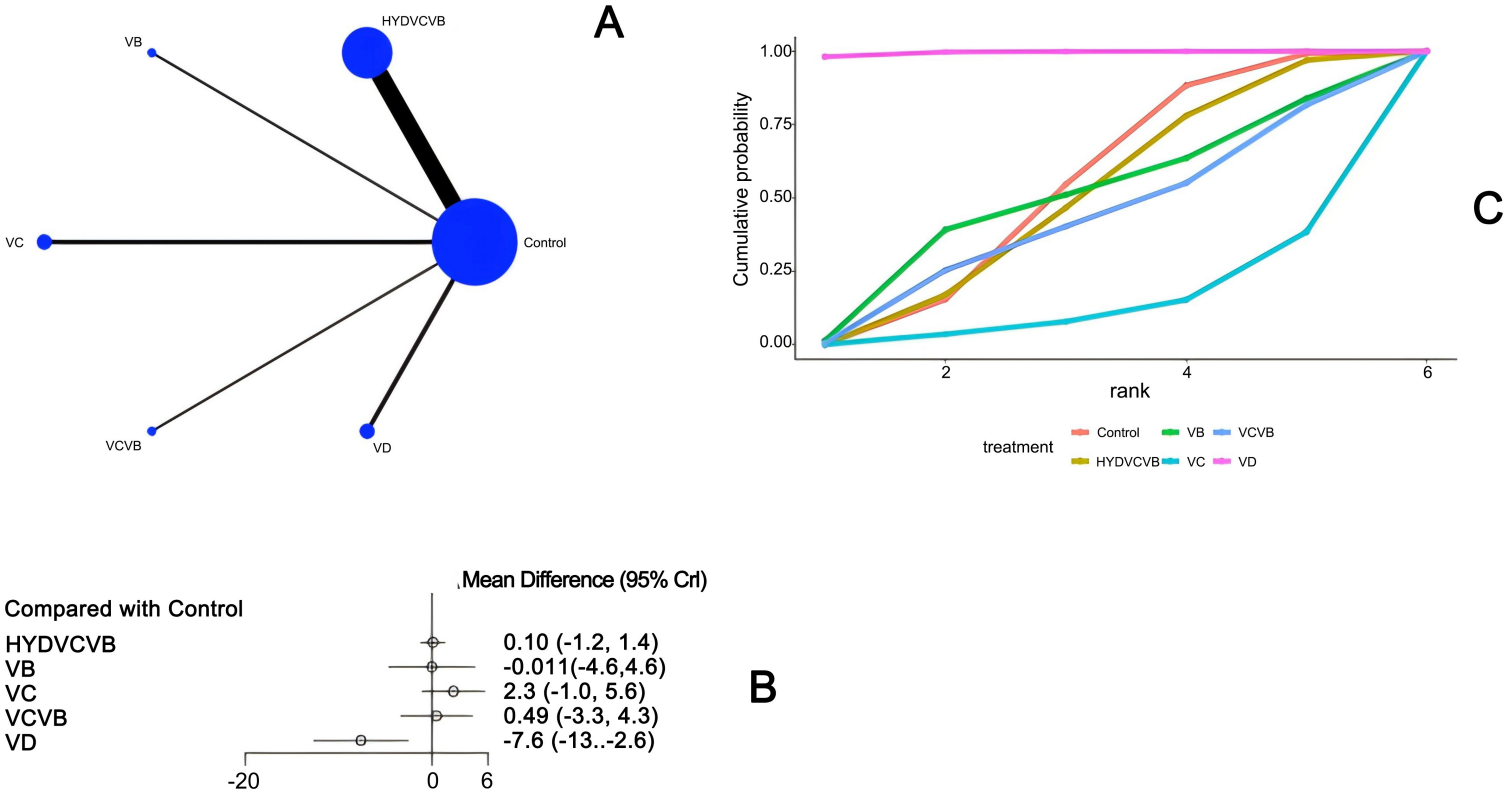
The effect of treatment on total length of hospital stay. **(A)** Network diagram illustrating the effect of treatment on total length of hospital stay. **(B)** Forest plot illustrating the effect of treatment on total length of hospital stay. **(C)** SUCRA plot illustrating the effect of treatment on total length of hospital stay.

Thirteen studies (20, 23, 26–28, 30, 39, 40, 44, 46, 47, 54) have examined the impact of different vitamin treatments on the total length of hospital stay, as shown in Figure 6. The network diagram reveals that direct comparisons were formed, with the most studies comparing HYDVCVB to the control group. The detailed network relationship diagram is presented in Figure 6A. In the forest plot analysis, vitamin D (VD) was found to significantly reduce the total length of hospital stay when compared to the control group [MD = −7.6, 95% CI (−13.0, −2.6)]. This indicates that VD can effectively shorten the total hospital stay. The specific forest plot is detailed in Figure 6B. The league table further highlights that VD has a significant advantage in reducing the total length of hospital stay when compared to the control group [MD = 7.61, 95% CI (2.59, 12.63)], HYDVCVB [MD = 7.71, 95% CI (2.55, 12.9)], vitamin B [MD = 7.6, 95% CI (0.84, 14.39)], vitamin C [MD = 9.93, 95% CI (3.9, 15.92)], and VCVB [MD = 8.1, 95% CI (1.79, 14.41)]. These differences are statistically significant, suggesting that VD is more effective in reducing the total length of hospital stay. The detailed league table is provided in Supplementary Table S4. The Surface Under the Cumulative Ranking Curve (SUCRA) plot shows that VD has the largest SUCRA value, indicating that VD may be the most effective treatment for reducing the total length of hospital stay in patients with septic shock. The SUCRA plot is detailed in Figure 6C.

#### 3.3.5 Effect of treatment on 28-day mortality

**Figure 7:**
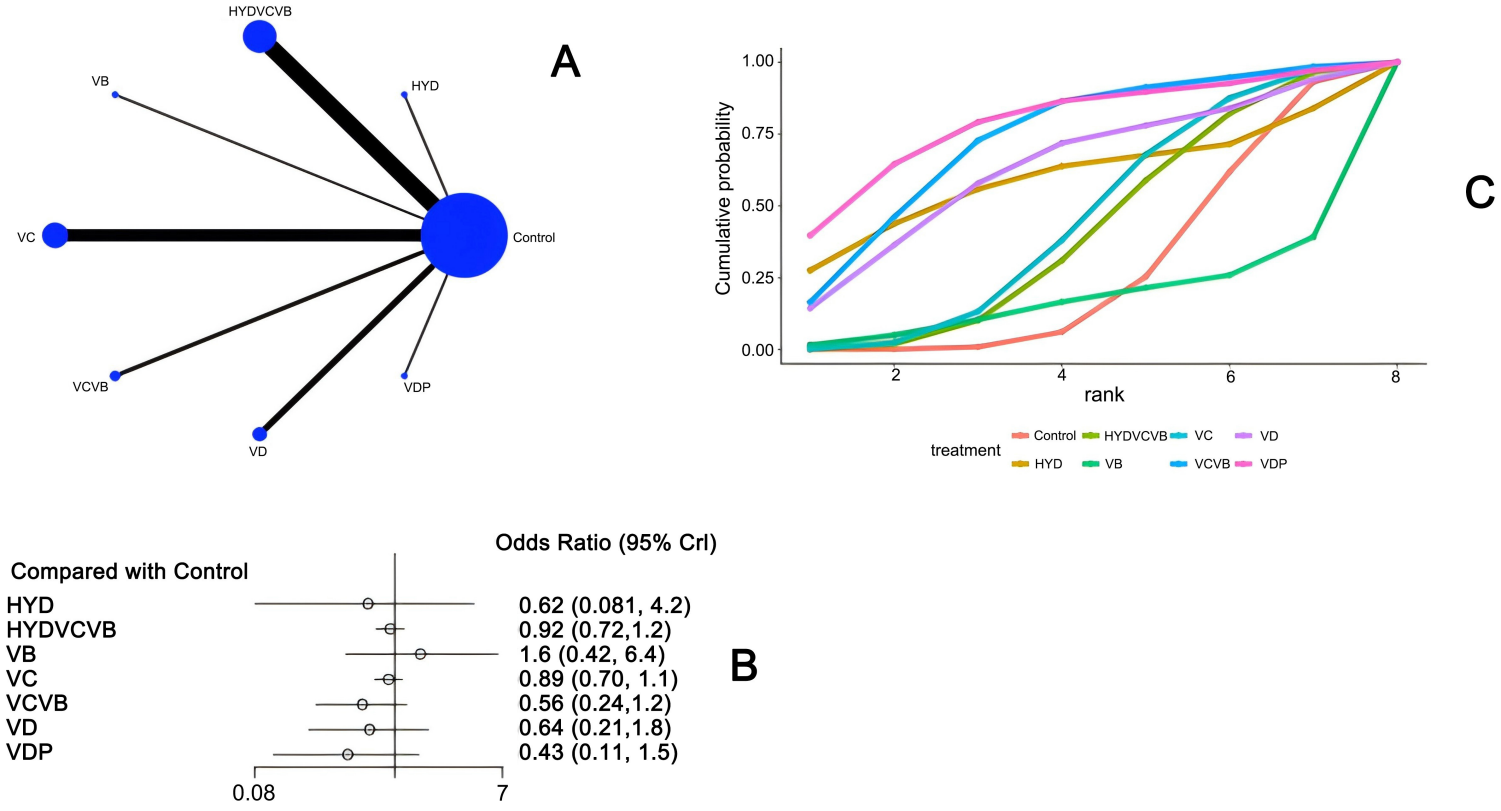
The effect of treatment on 28-day mortality. **(A)** Network diagram illustrating the effect of treatment on 28-day mortality. **(B)** Forest plot illustrating the effect of treatment on 28-day mortality. **(C)** SUCRA plot illustrating the effect of treatment on 28-day mortality.

Nineteen studies (20, 21, 26–28, 31, 32, 34, 35, 40–42, 44, 45, 47, 48, 52–54) have examined the impact of different vitamin treatments on 28-day mortality, as shown in Figure 7. The network diagram reveals that direct comparisons were formed, with the most studies comparing HYDVCVB to the control group. The detailed network relationship diagram is presented in Figure 7A. In the forest plot analysis, no significant differences were found when comparing HYD, HYDVCV, VB, VC, VCVB, VD, and VDP to the control group. The specific forest plot is detailed in Figure 7B. The league table further indicates that there were no statistically significant differences when comparing HYD, HYDVCV, VB, VC, VCVB, VD, and VDP to the control group. The detailed league table is provided in Supplementary Table S5. The Surface Under the Cumulative Ranking Curve (SUCRA) plot is shown in Figure 7C. In summary, the application of HYD, HYDVCV, VB, VC, VCVB, VD, and VDP in septic shock does not significantly affect 28-day mortality.

### 3.4 Publication bias assessment

A funnel graph was adopted to assess the publication bias of ICU duration, mechanical ventilation time, SOFA score, total hospital stay, and 28-day death rate. The findings indicated a high likelihood of publication bias in mechanical ventilation duration, as illustrated in Supplementary material S2 (Figures S1–S5).

## 4 Discussion

At present, several common vitamins (VA, VB, VC, VD, and VE) in the treatment of sepsis has been covered both domestically and internationally, but most studies compare the efficacy of a single vitamin to a control group. The evaluation and ranking of the effects of different vitamin interventions on sepsis patients are not clear. Therefore, this study uses the Bayesian network meta-analysis method to evaluate the impact of different vitamins on septic shock patients, which is the innovation of this paper.

Sepsis is a life-threatening organ dysfunction caused by a dysregulated host response to infection and is a major cause of high morbidity and mortality worldwide. Since there are no direct treatments targeting the pathogenesis of sepsis, clinical management relies on early recognition and prompt administration of antibiotics, intravenous fluids, and appropriate vasopressors (55). Over the past three decades, numerous epidemiological studies have shown a strong correlation between VD deficiency and the incidence of various infectious diseases, including sepsis (56–59). Therefore, the development of new adjuvant therapies can help improve disease prognosis or enhance therapeutic effects.

VD is a steroid hormone and a key nutrient that is reported to control a wide range of physiological processes (60). VD is available in several forms, existing as D2 and D3. In the body, D2 and D3 undergo two consecutive hydroxylation steps in the liver and are then converted into their active compounds in the kidneys, which are 25(OH)D3 calcidiol (a clinical marker of plasma VD levels) and 1,25(OH)2D3 calcitriol (61). Although there is no consensus in the literature on the plasma concentration of 25(OH)D used to define VD deficiency, it is a very common condition worldwide (62–64). Low plasma VD levels are observed in 79%−98% of intensive care unit patients, including sepsis cases (65–67). The risk of sepsis and its consequences (such as mortality, hospital stay, and organ failure) is positively correlated with VD deficiency (65, 68). Trongtrakul and Feemuchang found that three-quarters of patients diagnosed with severe sepsis had low plasma VD levels, with higher mortality rates, especially when VD plasma levels were severely deficient (25(OH)D < 30 nmol/L) (69). Therefore, restoring VD to optimal plasma levels may have an important impact on the development and outcome of sepsis. There are also studies showing that VD administration leads to a significant increase in the expression of the antimicrobial peptide cathelicidin (LL-37) (70) in white blood cell mRNA and plasma cathelicidin, a significant decrease in IL-1β and IL-6 in sepsis patients (54), and a reduction in 30-day ICU readmission rates in sepsis cases, a reduction in hospital mortality in critically ill patients with severe VD deficiency (25(OH)D3 ≤ 30 nmol/L) (71), a significant reduction in hospital stay (72), a reduction in mechanical ventilation time and hospital stay, and a reduction in mortality in critically ill patients in the ICU (73). This study shows that among septic shock patients, VD may be the most effective in reducing ICU hospital stay and total hospital stay.

A systematic review and meta-analysis of 27 studies, including 17 case-control studies and 10 cohort studies, found that the levels of 25-(OH)D in mothers and newborns with sepsis were significantly lower than those in non-septic children (*P* < 0.001). In addition, the proportion of severe VD deficiency in the sepsis group was significantly higher than in the non-sepsis group (OR = 2.66, 95% confidence interval CI = 1.13–6.25, *P* < 0.001). In this study, the incidence of sepsis in children with lower 25-(OH)D levels was 30.4%, while in children with higher 25-(OH)D levels it was 18.2%, but there were no statistically significant differences between the two groups in terms of mechanical ventilation rate and 30-day mortality (74). VD supplementation may become a new adjuvant therapy for sepsis in children. However, the current research results on the relationship between VD supplementation and the occurrence of sepsis in children are still controversial, and further research is needed to determine the role of VD supplementation in pediatric sepsis.

Sepsis patients often have intestinal dysbiosis, characterized by a decrease in beneficial bacteria and an increase in harmful bacteria. This imbalance can lead to impaired intestinal barrier function and increase the risk of systemic inflammatory responses (75). A study found that the abundance of Bifidobacterium and Lactobacillus in the gut microbiota of sepsis patients was significantly reduced, while the abundance of Bacteroides and Proteobacteria was significantly increased (54, 76). VD can modulate the composition of the gut microbiota through its active form 1,25-dihydroxyvitamin D3 (1,25-(OH)2D3). Studies have shown that the activation of the vitamin D receptor (VDR) can promote the growth of beneficial bacteria (such as Bifidobacterium and Lactobacillus) while inhibiting the proliferation of harmful bacteria (such as Bacteroides and Proteobacteria) (77, 78). In addition, VD reduces the production of inflammatory mediators by modulating the gut microbiota. Beneficial bacteria (such as Bifidobacterium and Lactobacillus) can produce short-chain fatty acids (SCFAs), which have anti-inflammatory effects and can modulate the function of immune cells to reduce inflammatory responses (79). A study found that VD supplementation can significantly increase the levels of SCFAs in the gut, thereby reducing the production of inflammatory mediators and improving the gut microenvironment (80)^.^ There are also studies that have found that early sepsis patients treated with a combination of probiotics [Winclove 607 based on Omnibiotic(R) 10 AAD] for 28 days did not change gut permeability, but endotoxins, endotoxin-binding proteins, and peptidoglycans increased. It can be seen that probiotic intervention successfully increased the probiotic strains in the feces and improved functional diversity (74). In severe sepsis children, supplementing with probiotics for 7 days can significantly reduce the levels of pro-inflammatory cytokines, increase anti-inflammatory cytokines, significantly reduce the sequential organ failure assessment score, but there is no significant improvement in mortality (75). Probiotics, as non-pathogenic microorganisms, have a positive impact on specific beneficial bacteria such as Lactobacillus, Bifidobacterium, and yeast, shaping the gut microbiota and restoring the composition of gut microbiota metabolites, reducing the susceptibility to sepsis (81–83). In addition, VD deficiency can lead to perturbations in the gut microbiome (84), and VD and VDR play an important role in maintaining the balance of the gut microbiota (85), which in turn enhances immunity to gut and systemic pathogens (86). Therefore, VD combined with probiotics can be a sepsis intervention method to restore balanced gut microbiota. This study shows that among septic shock patients, VD combined with probiotics may have an advantage in reducing the SOFA score after 24 h, and more high-quality randomized controlled trials are needed in the future to further verify its clinical value.

Cobalamin (vitamin B12) is an essential trace nutrient found in animal proteins, playing an important role in the function of the central nervous system and bone marrow (87). Vitamin B12 has anti-inflammatory and antioxidant properties and plays an important role in the pathophysiological process of sepsis (87–90). These effects specifically include: (1) selectively inhibiting inducible nitric oxide synthase, thereby reducing the production of nitric oxide. (2) Reducing the generation of reactive oxygen species by optimizing the use of glutathione. (3) Increasing the synthesis of acetylcholine and enhancing the function of the cholinergic anti-inflammatory pathway. (4) Promoting the process of oxidative phosphorylation. (5) Enhancing antibacterial capacity. (6) Regulating the activation of nuclear factor κB (NF-κB) (89, 91). Although cobalamin has many theoretical advantages and has shown good tolerance when administered intravenously in high doses for cyanide poisoning treatment (89, 91), its potential benefits in prospective clinical trials have not been confirmed and further exploration is needed.

In recent years, the potential role of VC in the treatment of septic shock has gradually received attention. VC is a powerful antioxidant and a cofactor for many biosynthetic enzymes, involved in the synthesis of endogenous vasopressin and norepinephrine (92). Since the human body cannot synthesize VC endogenously, and the serum VC levels of sepsis patients are usually low (93), supplementing VC has become a possible treatment option. Early studies have confirmed that intravenous VC (IVVC) is associated with reduced sepsis inflammatory response and improved outcomes (94, 95). However, the current research results on the efficacy of VC in treating sepsis or septic shock are not consistent. A meta-analysis including 18 randomized controlled trials with a total of 3,364 patients showed that IVVC treatment can significantly improve the ΔSOFA score and shorten the use of vasopressors, but it is not related to a reduction in short-term mortality (96). In addition, favorable outcomes have been reported in some meta-analyses for the VC group (97, 98). However, the existing evidence is still inconsistent. In another recent meta-analysis, intravenous VC seems to be ineffective in sepsis (99). The 2021 “Surviving Sepsis Campaign: International Guidelines for the Management of Sepsis and Septic Shock” suggests that for patients with sepsis or septic shock, the use of IVVC is not recommended and is only weakly recommended based on low-quality evidence (100).

In 2017, Paul Marik published for the first time the “sepsis cocktail” therapy (that is, the combined use of vitamin C, hydrocortisone, and thiamine), and reported its potential benefits in reducing mortality in sepsis patients, reducing the use of vasopressors, and reducing organ damage (95). This study was a single-center before-and-after study, and although the results were encouraging, it was also criticized mainly for the lack of support from randomized controlled trials (RCTs). In addition, multiple RCT studies and meta-analysis results have shown that the combination of vitamin C, hydrocortisone, and thiamine does not significantly improve mortality (44, 45, 101, 102).

Although this study explored the differences between different vitamins, we found in the league table that the differences between the top-ranked interventions are not obvious. Because for the selection of vitamins, we need more research to support our views, but this can also provide a treatment option for sepsis shock patients. Limitations of this study: all studies included in this study were in English, which may introduce some bias and affect the generalizability of the results. Secondly, some studies did not mention the method of random grouping, did not describe allocation concealment in detail, and did not mention the use of blinding. Therefore, more high-quality, large-scale, multicenter randomized controlled trials are needed to further verify the clinical effects of these interventions.

## 5 Conclusion

In patients with septic shock, the use of vitamin D shows certain advantages in reducing the number of days of ICU stay and the total length of hospital stay, and its combination with probiotics may help to lower the SOFA score after 24 h. However, these interventions have not significantly affected the 28-day mortality rate or the duration of mechanical ventilation.

## Data Availability

The original contributions presented in the study are included in the article/Supplementary material, further inquiries can be directed to the corresponding author.
