# Supplementary material for: Effects of different vitamins on individuals with septic shock: a Bayesian NMA of RCTs
Source: Front Nutr. 2025 Aug 13;12:1566422. doi: 10.3389/fnut.2025.1566422 (PMC12380562; doi:10.3389/fnut.2025.1566422)
Supplement: Supplementary file 1 [file Data_Sheet_1.docx]

Table S1.The league table of ICU length of stay

| MD 95%CI | | | | |
| --- | --- | --- | --- | --- |
| Control |  |  |  |  |
| -0.86 (-4.28, 2.89) | HYDVCVB |  |  |  |
| -1.01 (-5.85, 3.9) | -0.15 (-6.34, 5.77) | VB |  |  |
| -0.4 (-5.72, 4.74) | 0.49 (-6.17, 6.57) | 0.63 (-6.68, 7.63) | VC |  |
| 4.57 (1.01, 9.69)* | 5.4 (0.51, 11.66)* | 5.59 (-0.1, 12.88) | 4.92 (-0.91, 12.69) | VD |

*means P<0.05

Table S2. The league table of mechanical ventilation duration

| MD 95%CI | | | | |
| --- | --- | --- | --- | --- |
| Control |  |  |  |  |
| 0.64 (-0.48, 1.76) | HYDVCVB |  |  |  |
| 4.99 (-9.2, 19.16) | 4.35 (-9.88, 18.56) | VC |  |  |
| 0.99 (-1.04, 3.04) | 0.36 (-1.97, 2.68) | -4.01 (-18.32, 10.34) | VCVB |  |
| 1.26 (-2.55, 5.07) | 0.61 (-3.35, 4.57) | -3.73 (-18.43, 10.95) | 0.26 (-4.06, 4.59) | VD |

Table S3. The league table of SOFA scores after 24 hours

| MD 95%CI | | | | | | | | |
| --- | --- | --- | --- | --- | --- | --- | --- | --- |
| Control |  |  |  |  |  |  |  |  |
| 0.8 (-0.43, 2.02) | HYDVC |  |  |  |  |  |  |  |
| -0.32 (-0.88, 0.06) | -1.12 (-2.53, 0.11) | HYDVCVB |  |  |  |  |  |  |
| 0.02 (-0.59, 0.66) | -0.78 (-2.14, 0.62) | 0.34 (-0.35, 1.23) | VB |  |  |  |  |  |
| 0.07 (-0.31, 0.48) | -0.72 (-2.01, 0.58) | 0.39 (-0.11, 1.11) | 0.05 (-0.69, 0.78) | VC |  |  |  |  |
| -0.21 (-0.97, 0.73) | -1.01 (-2.38, 0.6) | 0.12 (-0.74, 1.3) | -0.22 (-1.2, 0.9) | -0.28 (-1.12, 0.74) | VCVB |  |  |  |
| 0.07 (-0.93, 1.06) | -0.73 (-2.29, 0.84) | 0.37 (-0.58, 1.59) | 0.06 (-1.14, 1.19) | 0 (-1.08, 1.04) | 0.29 (-1.16, 1.41) | VD |  |  |
| 2.98 (0.27, 5.62)* | 2.18 (-0.79, 5.1) | 3.32 (0.59, 6.04)* | 2.96 (0.18, 5.67)* | 2.91 (0.17, 5.57)* | 3.18 (0.31, 5.9)* | 2.91 (0.05, 5.71)* | VDP |  |
| 0.27 (-0.94, 1.47) | -0.53 (-2.26, 1.19) | 0.6 (-0.63, 1.97) | 0.25 (-1.13, 1.59) | 0.2 (-1.09, 1.45) | 0.48 (-1.12, 1.84) | 0.2 (-1.36, 1.74) | -2.72 (-5.62, 0.24) | VE |

Table S4. The league table of length of stay in hospital

| MD 95%CI | | | | | |
| --- | --- | --- | --- | --- | --- |
| Control |  |  |  |  |  |
| -0.1 (-1.37, 1.17) | HYDVCVB |  |  |  |  |
| 0.01 (-4.56, 4.57) | 0.11 (-4.64, 4.83) | VB |  |  |  |
| -2.31 (-5.62, 1.01) | -2.2 (-5.76, 1.35) | -2.31 (-7.95, 3.32) | VC |  |  |
| -0.49 (-4.31, 3.3) | -0.38 (-4.4, 3.63) | -0.5 (-6.42, 5.42) | 1.82 (-3.23, 6.85) | VCVB |  |
| 7.61 (2.59, 12.63)* | 7.71 (2.55, 12.9)* | 7.6 (0.84, 14.39)* | 9.93 (3.9, 15.92)* | 8.1 (1.79, 14.41)* | VD |

Table S5. The league table of 28-day mortality

| OR 95%CI | | | | | | | |
| --- | --- | --- | --- | --- | --- | --- | --- |
| Control |  |  |  |  |  |  |  |
| 1.62 (0.24, 12.33) | HYD |  |  |  |  |  |  |
| 1.09 (0.85, 1.4) | 0.67 (0.09, 4.58) | HYDVCVB |  |  |  |  |  |
| 0.63 (0.16, 2.4) | 0.38 (0.03, 4.01) | 0.58 (0.14, 2.25) | VB |  |  |  |  |
| 1.12 (0.88, 1.43) | 0.69 (0.09, 4.73) | 1.03 (0.73, 1.46) | 1.78 (0.46, 7.31) | VC |  |  |  |
| 1.79 (0.81, 4.11) | 1.11 (0.13, 8.86) | 1.65 (0.72, 3.92) | 2.87 (0.6, 14.35) | 1.6 (0.7, 3.81) | VCVB |  |  |
| 1.57 (0.55, 4.68) | 0.97 (0.1, 8.75) | 1.45 (0.49, 4.44) | 2.52 (0.46, 14.49) | 1.41 (0.48, 4.31) | 0.88 (0.23, 3.38) | VD |  |
| 2.33 (0.65, 8.87) | 1.45 (0.13, 14.78) | 2.15 (0.58, 8.34) | 3.73 (0.59, 25.5) | 2.09 (0.57, 8.12) | 1.3 (0.28, 6.16) | 1.49 (0.28, 8.03) | VDP |


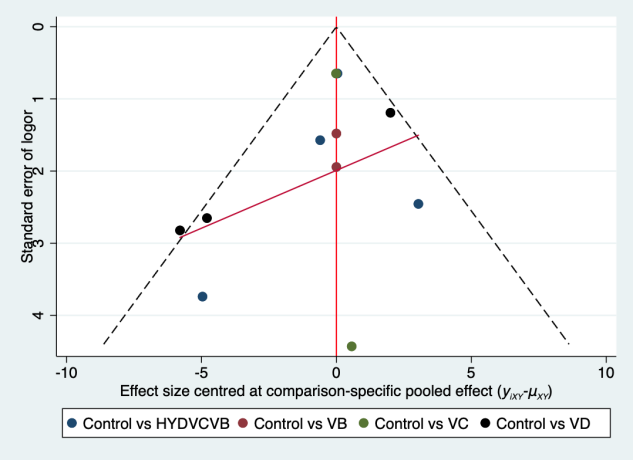


Figure S1. The funnel plot of ICU length of stay


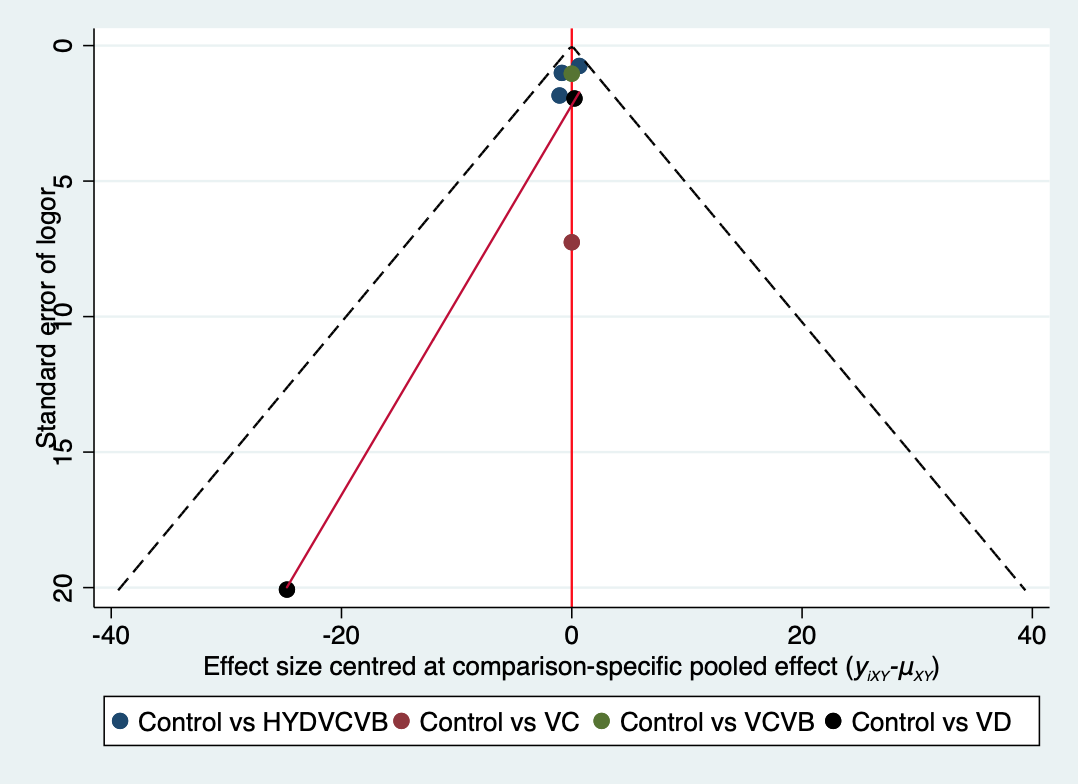


Figure S2. The funnel plot of mechanical ventilation duration


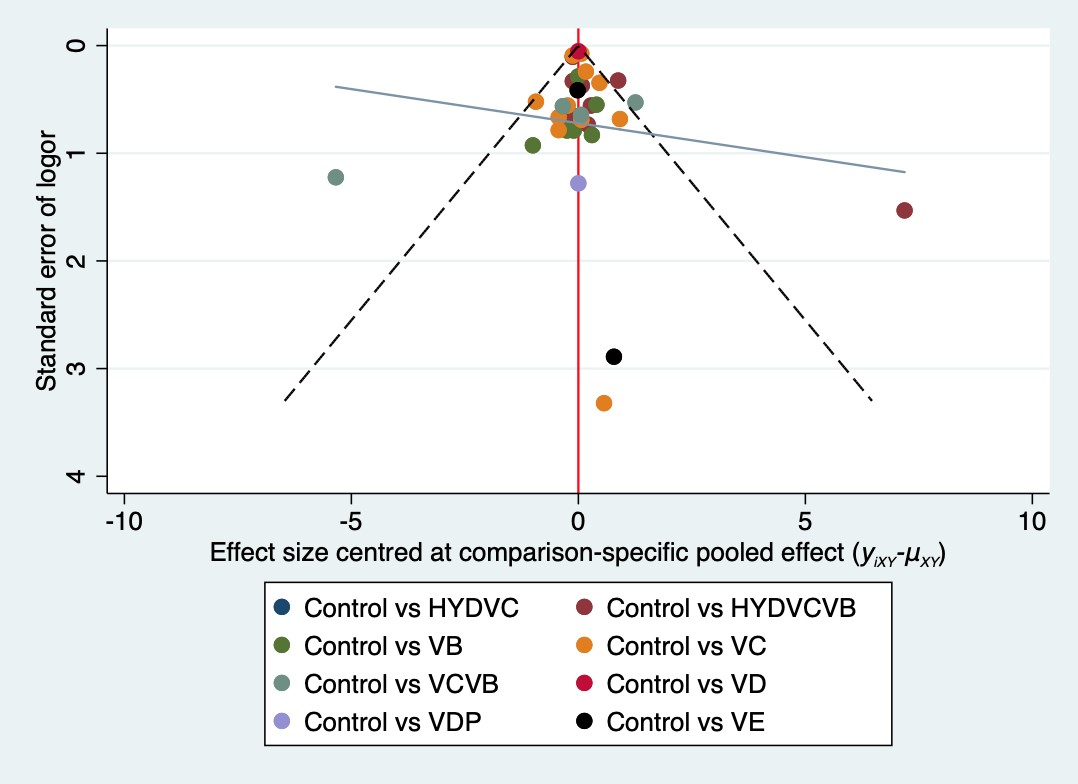


Figure S3. The funnel plot of SOFA scores after 24 hours


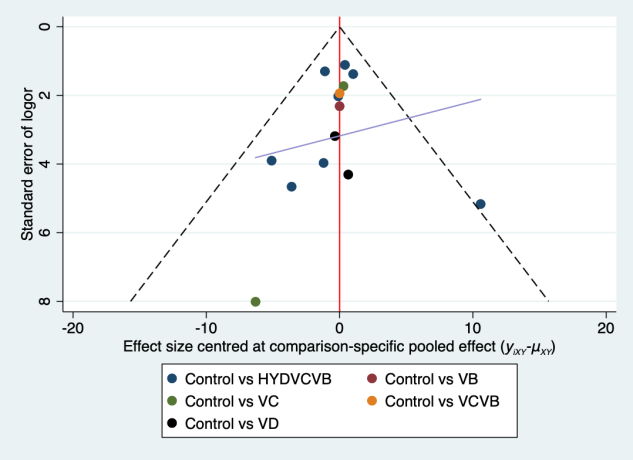


Figure S4. The funnel plot of length of stay in hospital


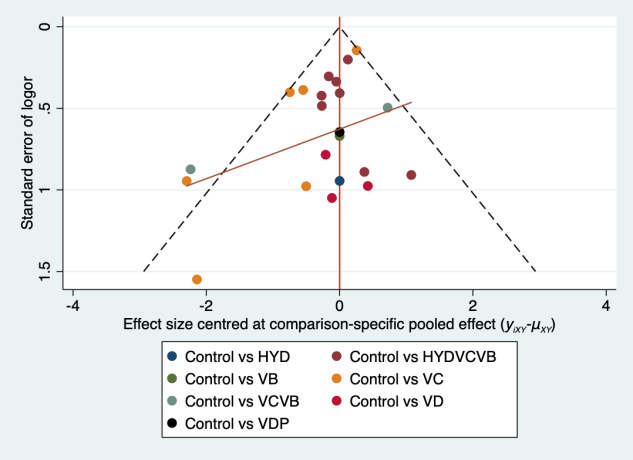


Figure S5. The funnel plot of 28-day mortality
