# Supplementary material for: Effects of different vitamins on individuals with septic shock: a Bayesian NMA of RCTs
Source: Front Nutr. 2025 Aug 13;12:1566422. doi: 10.3389/fnut.2025.1566422 (PMC12380562; doi:10.3389/fnut.2025.1566422)
Supplement: Supplementary file 2 [file Table_1.DOCX]

Table S1. Search history of Pubmed

Shock, Septic[MeSH Terms] (((((((((((Shock, Septic[Title/Abstract]) OR (Endotoxin Shock[Title/Abstract])) OR (Endotoxin Shocks[Title/Abstract])) OR (Septic Shock[Title/Abstract])) OR (Shock Syndrome, Toxic[Title/Abstract])) OR (Shock, Endotoxic[Title/Abstract])) OR (Shock, Endotoxin[Title/Abstract])) OR (Shock, Toxic[Title/Abstract])) OR (Shocks, Endotoxin[Title/Abstract])) OR (Toxic Shock[Title/Abstract])) OR (Toxic Shock Syndrome[Title/Abstract])) OR (Toxic Shock Syndromes[Title/Abstract])(Shock, Septic[MeSH Terms]) OR ((((((((((((Shock, Septic[Title/Abstract]) OR (Endotoxin Shock[Title/Abstract])) OR (Endotoxin Shocks[Title/Abstract])) OR (Septic Shock[Title/Abstract])) OR (Shock Syndrome, Toxic[Title/Abstract])) OR (Shock, Endotoxic[Title/Abstract])) OR (Shock, Endotoxin[Title/Abstract])) OR (Shock, Toxic[Title/Abstract])) OR (Shocks, Endotoxin[Title/Abstract])) OR (Toxic Shock[Title/Abstract])) OR (Toxic Shock Syndrome[Title/Abstract])) OR (Toxic Shock Syndromes[Title/Abstract]))

(((((vitamins[MeSH Terms]) OR (vitamin A[MeSH Terms])) OR (Vitamin B Complex[MeSH Terms])) OR (vitamin C[MeSH Terms])) OR (vitamin D[MeSH Terms])) OR (vitamin E[MeSH Terms]) ((((((((((((((((((((((((((vitamins[Title/Abstract]) OR (vitamin[Title/Abstract])) OR (vitamin A[Title/Abstract])) OR (11-cis-Retinol[Title/Abstract])) OR (All Trans Retinol[Title/Abstract])) OR (All-Trans-Retinol[Title/Abstract])) OR (Aquasol A[Title/Abstract])) OR (Retinol[Title/Abstract])) OR (Vitamin A1[Title/Abstract])) OR (Vitamin B Complex[Title/Abstract])) OR (B Vitamins[Title/Abstract])) OR (B Vitamin[Title/Abstract])) OR (Vitamin, B[Title/Abstract])) OR (Neurobion[Title/Abstract])) OR (Vitamin B6[Title/Abstract])) OR (Vitamin B 6[Title/Abstract])) OR (Vitamin B 12[Title/Abstract])) OR (B 12, Vitamin[Title/Abstract])) OR (Vitamin B12[Title/Abstract])) OR (B12, Vitamin[Title/Abstract])) OR (Cyanocobalamin[Title/Abstract])) OR (Cobalamins[Title/Abstract])) OR (Cobalamin[Title/Abstract])) OR (Eritron[Title/Abstract])) OR (vitamin C[Title/Abstract])) OR (vitamin D[Title/Abstract])) OR (vitamin E[Title/Abstract]) ((((((vitamins[MeSH Terms]) OR (vitamin A[MeSH Terms])) OR (Vitamin B Complex[MeSH Terms])) OR (vitamin C[MeSH Terms])) OR (vitamin D[MeSH Terms])) OR (vitamin E[MeSH Terms])) OR (((((((((((((((((((((((((((vitamins[Title/Abstract]) OR (vitamin[Title/Abstract])) OR (vitamin A[Title/Abstract])) OR (11-cis-Retinol[Title/Abstract])) OR (All Trans Retinol[Title/Abstract])) OR (All-Trans-Retinol[Title/Abstract])) OR (Aquasol A[Title/Abstract])) OR (Retinol[Title/Abstract])) OR (Vitamin A1[Title/Abstract])) OR (Vitamin B Complex[Title/Abstract])) OR (B Vitamins[Title/Abstract])) OR (B Vitamin[Title/Abstract])) OR (Vitamin, B[Title/Abstract])) OR (Neurobion[Title/Abstract])) OR (Vitamin B6[Title/Abstract])) OR (Vitamin B 6[Title/Abstract])) OR (Vitamin B 12[Title/Abstract])) OR (B 12, Vitamin[Title/Abstract])) OR (Vitamin B12[Title/Abstract])) OR (B12, Vitamin[Title/Abstract])) OR (Cyanocobalamin[Title/Abstract])) OR (Cobalamins[Title/Abstract])) OR (Cobalamin[Title/Abstract])) OR (Eritron[Title/Abstract])) OR (vitamin C[Title/Abstract])) OR (vitamin D[Title/Abstract])) OR (vitamin E[Title/Abstract])) ((Shock, Septic[MeSH Terms]) OR ((((((((((((Shock, Septic[Title/Abstract]) OR (Endotoxin Shock[Title/Abstract])) OR (Endotoxin Shocks[Title/Abstract])) OR (Septic Shock[Title/Abstract])) OR (Shock Syndrome, Toxic[Title/Abstract])) OR (Shock, Endotoxic[Title/Abstract])) OR (Shock, Endotoxin[Title/Abstract])) OR (Shock, Toxic[Title/Abstract])) OR (Shocks, Endotoxin[Title/Abstract])) OR (Toxic Shock[Title/Abstract])) OR (Toxic Shock Syndrome[Title/Abstract])) OR (Toxic Shock Syndromes[Title/Abstract]))) AND (((((((vitamins[MeSH Terms]) OR (vitamin A[MeSH Terms])) OR (Vitamin B Complex[MeSH Terms])) OR (vitamin C[MeSH Terms])) OR (vitamin D[MeSH Terms])) OR (vitamin E[MeSH Terms])) OR (((((((((((((((((((((((((((vitamins[Title/Abstract]) OR (vitamin[Title/Abstract])) OR (vitamin A[Title/Abstract])) OR (11-cis-Retinol[Title/Abstract])) OR (All Trans Retinol[Title/Abstract])) OR (All-Trans-Retinol[Title/Abstract])) OR (Aquasol A[Title/Abstract])) OR (Retinol[Title/Abstract])) OR (Vitamin A1[Title/Abstract])) OR (Vitamin B Complex[Title/Abstract])) OR (B Vitamins[Title/Abstract])) OR (B Vitamin[Title/Abstract])) OR (Vitamin, B[Title/Abstract])) OR (Neurobion[Title/Abstract])) OR (Vitamin B6[Title/Abstract])) OR (Vitamin B 6[Title/Abstract])) OR (Vitamin B 12[Title/Abstract])) OR (B 12, Vitamin[Title/Abstract])) OR (Vitamin B12[Title/Abstract])) OR (B12, Vitamin[Title/Abstract])) OR (Cyanocobalamin[Title/Abstract])) OR (Cobalamins[Title/Abstract])) OR (Cobalamin[Title/Abstract])) OR (Eritron[Title/Abstract])) OR (vitamin C[Title/Abstract])) OR (vitamin D[Title/Abstract])) OR (vitamin E[Title/Abstract])))

Table S2. Search history of Cochrane

ID Search Hits

#1 MeSH descriptor: [Shock, Septic] explode all trees 1434

#2 (Shock, Septic):ti,ab,kw OR (Endotoxin Shock):ti,ab,kw OR (Endotoxin Shocks):ti,ab,kw OR (Septic Shock):ti,ab,kw OR (Shock Syndrome, Toxic):ti,ab,kw 4334

#3 (Shock, Endotoxic):ti,ab,kw OR (Shock, Endotoxin):ti,ab,kw OR (Shock, Toxic):ti,ab,kw OR (Shocks, Endotoxin):ti,ab,kw OR (Toxic Shock):ti,ab,kw 352

#4 (Toxic Shock Syndrome):ti,ab,kw OR (Toxic Shock Syndromes):ti,ab,kw 98

#5 #1 or #2 or #3 or #4 4382

#6 MeSH descriptor: [Vitamins] explode all trees 5960

#7 MeSH descriptor: [Vitamin A] explode all trees 2608

#8 MeSH descriptor: [Vitamin B Complex] explode all trees 1205

#9 MeSH descriptor: [Ascorbic Acid] explode all trees 2831

#10 MeSH descriptor: [Vitamin D] explode all trees 7967

#11 MeSH descriptor: [Vitamin E] explode all trees 3147

#12 (vitamins):ti,ab,kw OR (vitamin):ti,ab,kw OR (vitamin A):ti,ab,kw OR (cis Retinol):ti,ab,kw OR (All Trans Retinol):ti,ab,kw 39313

#13 (All-Trans-Retinol):ti,ab,kw OR (Aquasol A):ti,ab,kw OR (Retinol):ti,ab,kw OR (Vitamin A1):ti,ab,kw OR (Vitamin B Complex):ti,ab,kw 3797

#14 (B Vitamins):ti,ab,kw OR (B Vitamin):ti,ab,kw OR (Vitamin, B):ti,ab,kw OR (Neurobion):ti,ab,kw OR (Vitamin B6):ti,ab,kw 7346

#15 (Vitamin B 6):ti,ab,kw OR (Vitamin B 12):ti,ab,kw OR (B 12, Vitamin):ti,ab,kw OR (Vitamin B12):ti,ab,kw OR (B12, Vitamin):ti,ab,kw 5814

#16 (Cyanocobalamin):ti,ab,kw OR (Cobalamins):ti,ab,kw OR (Cobalamin):ti,ab,kw OR (Eritron):ti,ab,kw OR (vitamin C):ti,ab,kw 9671

#17 (vitamin D):ti,ab,kw OR (vitamin E):ti,ab,kw 23876

#18 #6 or #7 or #8 or #9 or #10 or #11 or #12 or #13 or #14 or #15 or #16 or #17 42982

#19 #5 and #18 184

Table S3. Search history of Web of science

| # | Search Query | Results |
| --- | --- | --- |
| 1 | TS=(Shock, Septic) OR TS=(Endotoxin Shock) OR TS=(Endotoxin Shocks) OR TS=(Septic Shock) OR TS=(Shock Syndrome, Toxic) OR TS=(Shock, Endotoxic) OR TS=(Shock, Endotoxin) OR TS=(Shock, Toxic) OR TS=(Shocks, Endotoxin) OR TS=(Toxic Shock) OR TS=(Toxic Shock Syndrome) OR TS=(Toxic Shock Syndromes) | 64746 |
| 2 | TS=(vitamins) OR TS=(vitamin) OR TS=(vitamin A) OR TS=(11-cis-Retinol) OR TS=(All Trans Retinol) OR TS=(All-Trans-Retinol) OR TS=(Aquasol A) OR TS=(Retinol) OR TS=(Vitamin A1) OR TS=(Vitamin B Complex) OR TS=(B Vitamins) OR TS=(B Vitamin) OR TS=(Vitamin, B) OR TS=(Neurobion) OR TS=(Vitamin B6) OR TS=(Vitamin B 6) OR TS=(Vitamin B 12) OR TS=(B 12, Vitamin) OR TS=(Vitamin B12) OR TS=(B12, Vitamin) OR TS=(Cyanocobalamin) OR TS=(Cobalamins) OR TS=(Cobalamin) OR TS=(Eritron) OR TS=(vitamin C) OR TS=(vitamin D) OR TS=(vitamin E) | 369828 |
| 3 | #2 AND #1 | 748 |

Table S4. Search history of Embase

| No. | Query | Results |
| --- | --- | --- |
| #49 | #14 AND #48 | 1935 |
| #48 | #15 OR #16 OR #17 OR #18 OR #19 OR #20 OR #21 OR #22 OR #23 OR #24 OR #25 OR #26 OR #27 OR #28 OR #29 OR #30 OR #31 OR #32 OR #33 OR #34 OR #35 OR #36 OR #37 OR #38 OR #39 OR #40 OR #41 OR #42 OR #43 OR #44 OR #45 OR #46 OR #47 | 921612 |
| #47 | 'vitamin e':ab,ti | 38591 |
| #46 | 'vitamin d':ab,ti | 126870 |
| #45 | 'vitamin c':ab,ti | 33930 |
| #44 | 'eritron':ab,ti | 1 |
| #43 | 'cobalamin':ab,ti | 6238 |
| #42 | 'cobalamins':ab,ti | 483 |
| #41 | 'cyanocobalamin':ab,ti | 1935 |
| #40 | 'b12, vitamin':ab,ti | 774 |
| #39 | 'vitamin b12':ab,ti | 22083 |
| #38 | 'b 12, vitamin':ab,ti | 302 |
| #37 | 'vitamin b 12':ab,ti | 13019 |
| #36 | 'vitamin b 6':ab,ti | 4397 |
| #35 | 'vitamin b6':ab,ti | 6767 |
| #34 | 'neurobion':ab,ti | 38 |
| #33 | 'vitamin, b':ab,ti | 21501 |
| #32 | 'b vitamin':ab,ti | 1972 |
| #31 | 'b vitamins':ab,ti | 3754 |
| #30 | 'vitamin b complex':ab,ti | 981 |
| #29 | 'vitamin a1':ab,ti | 59 |
| #28 | 'retinol':ab,ti | 19073 |
| #27 | 'aquasol a':ab,ti | 9 |
| #26 | 'all-trans-retinol':ab,ti | 834 |
| #25 | 'all trans retinol':ab,ti | 834 |
| #24 | '11-cis-retinol':ab,ti | 273 |
| #23 | 'vitamin a':ab,ti | 32609 |
| #22 | 'vitamin':ab,ti | 317662 |
| #21 | 'vitamins':ab,ti | 49535 |
| #20 | 'alpha tocopherol'/exp | 82899 |
| #19 | 'vitamin d'/exp | 193716 |
| #18 | 'ascorbic acid'/exp | 124465 |
| #17 | 'vitamin b complex'/exp | 6733 |
| #16 | 'retinol'/exp | 52880 |
| #15 | 'vitamin'/exp | 844919 |
| #14 | #1 OR #2 OR #3 OR #4 OR #5 OR #6 OR #7 OR #8 OR #9 OR #10 OR #11 OR #12 OR #13 | 87881 |
| #13 | 'toxic shock syndromes':ab,ti | 44 |
| #12 | 'toxic shock syndrome':ab,ti | 5081 |
| #11 | 'toxic shock':ab,ti | 6196 |
| #10 | 'shocks, endotoxin':ab,ti | 0 |
| #9 | 'shock, toxic':ab,ti | 26 |
| #8 | 'shock, endotoxin':ab,ti | 46 |
| #7 | 'shock, endotoxic':ab,ti | 20 |
| #6 | 'shock syndrome, toxic':ab,ti | 6 |
| #5 | 'septic shock':ab,ti | 47650 |
| #4 | 'endotoxin shocks':ab,ti | 7 |
| #3 | 'endotoxin shock':ab,ti | 2333 |
| #2 | 'shock, septic':ab,ti | 213 |
| #1 | 'septic shock'/exp | 77977 |
